# Evaluation of Structural and Compositional Changes of a Model Monoaromatic Hydrocarbon in a Benchtop Hydrocracker Using GC, FTIR, and NMR Spectroscopy

**DOI:** 10.1021/acsomega.3c03833

**Published:** 2023-09-18

**Authors:** Debashis Puhan, Michael T. L. Casford, Paul B. Davies

**Affiliations:** Yusuf Hamied Department of Chemistry, University of Cambridge, Lensfield Road, Cambridge CB2 1EW, United Kingdom

## Abstract

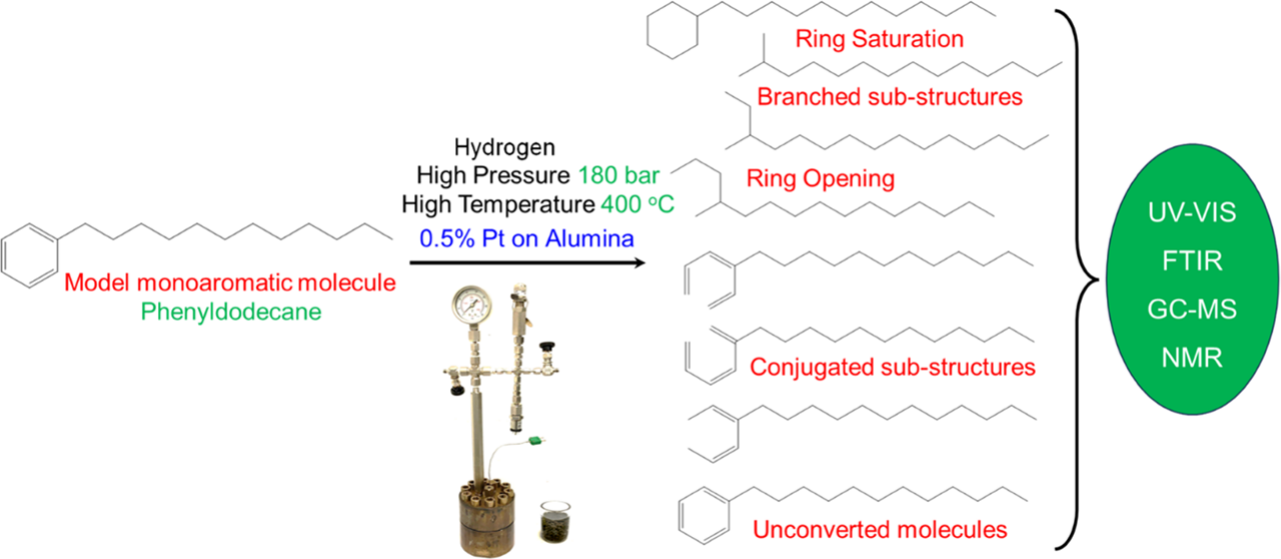

Hydrogenation is a catalytic process that has the potential to facilitate sustainable chemical production. In this work, a model monoaromatic hydrocarbon, phenyldodecane (PDD), comprising an aromatic ring with a long aliphatic side chain has been chosen as representative of a typical species involved in hydrogenation and hydrocracked at a high pressure and temperature over a platinum catalyst in a bespoke benchtop mini-reactor. Gas chromatography–mass spectrometry (GC–MS), Fourier transform infrared (FTIR) spectroscopy, UV–vis spectroscopy, and nuclear magnetic resonance (NMR) spectroscopy were employed to analyze the changes that took place after hydrocracking for different time periods. By combining the results from these sensitive spectroscopic tools, it was found that along with the saturation of the aromatic ring of PDD by hydrogen addition, new molecules were formed via ring opening and catalytic cracking. For comparison purposes, the spectra of the samples post hydrogenation were compared with those of cyclohexylnonadecane (CHND), which has a saturated six-membered ring and a long aliphatic tail.

## 1 Introduction

The starting materials required to produce a plethora of consumer products such as clothes, tires, detergents, solvents, digital devices, and countless other items in daily use are petrochemicals. Furthermore, fundamentally important chemicals and pharmaceuticals are produced from feedstock derived from the distillation of crude oil, which is not only a nonrenewable resource but also a major contributor to global warming. The implementation of a sustainable means of chemical production has thus become a key objective to meet essential sustainability goals. To achieve these goals, it is immensely important to increase the energy efficiency required for petrochemical production and to explore potential options for making petrochemically sourced products in a sustainable manner.

Chemical processes such as hydrogenation and dehydrogenation are essential methods used for the synthesis of chemicals, fuels, and edible fats from petrochemicals.^1^ These processes operate at elevated pressures and temperatures and require a suitable catalyst. In hydrogenation, the objective is to control the hydrogen-to-carbon (H/C) ratio in the desired products. Controlling the H/C ratio of the products requires either lowering the C content of the products by carbon rejection or increasing the H content by hydrogenation. In practice, the hydrogen addition route comes at a greater cost than carbon rejection because producing hydrogen and hydrogenation catalysts are both very expensive. However, as the production capacity of “green” hydrogen continues to increase, hydrogenation becomes a much more viable option.

In practice, hydrogenation or hydrogen addition is implemented using a refining technology known as hydroprocessing. Hydroprocessing is a generic term covering various processes such as hydrotreating, hydrocracking, hydrodesulfurization, hydro-dewaxing, and hydroisomerization, which are selected depending on the severity of the treatment required to achieve the desired product. Various hydroprocessing technologies have been employed in both coal liquefaction and gas-to-liquid conversion for producing liquid hydrocarbons,^2^ producing sustainable bio-fuels^3^ and lubricant base stocks.^4−7^ In addition, hydrogenation is also used in food production, e.g., margarine; specifically, hydrogen is added to unsaturated hydrocarbon molecules to solidify them and make them more spreadable and easier for shaping and packaging.

Additionally, as catalysts play an important role in increasing yields and improving selectivity,^8^ the catalysts required for hydrogenation should themselves be recyclable for environmental reasons. An extensive understanding of catalyst performance and the optimum physicochemical conditions they require can be achieved with small-scale reactors. This data can be used to optimize the composition and yield of the product for a particular process and catalyst so that it gives the best performance before scaling up.

In this work, we present the results from a simple prototype benchtop hydrogenation reactor operating at elevated temperatures and pressures. A monoaromatic molecule with a long aliphatic side chain, phenyldodecane (PDD), was chosen as a model to study the changes occurring during hydrogenation. Routine quality control protocols are based on readily measured physical properties such as density, refractive index (RI), solubility, and freezing and boiling points. This data is not sufficiently detailed for obtaining specific knowledge on composition and formulation, which is available from high-resolution analytical techniques such as gas chromatography–mass spectroscopy (GC–MS), Fourier transform infrared (FTIR) spectroscopy, and nuclear magnetic resonance (NMR) spectroscopy.

This article demonstrates the usefulness of these techniques for identifying the chemical changes occurring when using a small-scale benchtop hydrocracker reactor. Although the expected changes such as ring opening, carbon–carbon bond fission, and isomerization of paraffins have been proposed earlier, the literature on quantifying chemical change using a range of analytical chemistry techniques based on spectroscopic measurements is sparse. Hydrogenation of the aromatic ring of PDD as well as hydrocracking of its aliphatic tail is also anticipated. To throw more light on this process, we have also examined cyclohexane nonadecane (CHND), which has a saturated six-membered ring, for comparison purposes.

## 2 Materials and Methods

**Figure 1:**
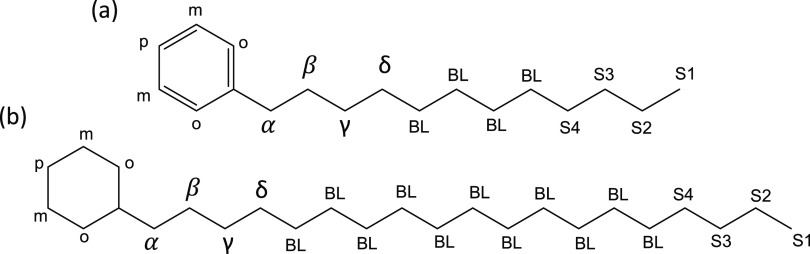
Structures of (a) 1-phenyldodecane (PDD) and (b) cyclohexylnonadecane (CHND) with their carbon atom labeling.

1-Phenyldodecane (Merck, Germany) and cyclohexylnonadecane (Tokyo Chemical Industry UK Ltd.) were used as supplied. The platinum (Pt) on alumina catalyst (Merck, Germany) loaded with 0.5% Pt was in the form of 1.5 mm diameter spheres. The structures of 1-phenyldodecane (C_18_H_30_) and cyclohexylnonadecane (C_25_H_50_) are shown in Figure 1 with their carbon positions labeled as follows: branchless carbons, BL, the four terminal carbons in the aliphatic chain S1, S2, S3, and S4, and with α, β, γ, and δ carbon positions closest to the ring. The latter four are specifically distinguished from the others because they are most affected by bonding changes occurring in the ring.

**Figure 2:**
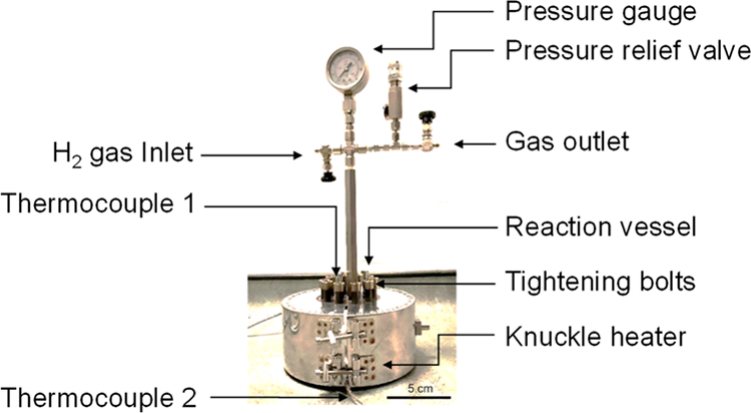
Benchtop hydrocracker with a 5 cm scale bar shown. The 30 mL reactor vessel is completely enclosed within the heater.

The PDD samples were hydrogenated using a 30 mL capacity benchtop hydrocracker reactor constructed in house (Figure 2). It can withstand pressures up to 275 bar and temperatures up to 500 °C. 10 g of sample and 1 g of catalyst were used for each hydrogenation and thoroughly premixed before the reactor was loaded and run for 2, 4, or 8 h. The hydrocracker was first filled to 120 bars with high-purity hydrogen at room temperature (16–18 °C). At the set temperature of 400 °C, the maximum pressure reached is about 180 bars. The reactor takes about 40 min to reach 400 °C from room temperature. As the reaction progresses, the hydrogen gas is consumed and consequently the pressure gradually drops to 110–120 bars. The hydrocracker has a residual pressure of 60–70 bars at room temperature, and after depressurizing, it is opened to retrieve the liquid samples. The samples collected at each stage were analyzed using refractive index, FTIR, UV–vis, NMR, and GC–MS measurements. The sample labeling nomenclature is given in Table 1. Throughout this paper, we have used the terms hydrogenation and hydrocracking interchangeably as hydrogenation at high temperature and pressure also causes catalytic cracking.^9^

**Table 1:** Sample Nomenclature

PDD	phenyldodecane
2 h HC	hydrocracked for 2 h
4 h HC	hydrocracked for 4 h
8 h HC	hydrocracked for 8 h

Refractive indices were measured using a Kern Abbe analogue refractometer at 20 °C. Uncertainty in the measurement is ± 0.0005 units. An average of 5 measurements are reported. Repeat measurements are a combination of readings from repeat hydrogenation sample runs as well as same sample on different days.

UV–vis measurements of samples were carried out on a PerkinElmer (Lambda 25) UV–vis spectrometer using a rectangular quartz cuvette with a 1 mm path length and 350 μL volume. The scans were recorded at 1 nm resolution at a speed of 480 nm/min. The visible-to-UV lamp change occurs at 326 nm.

Attenuated total reflectance (ATR)–FTIR spectra were recorded at 4 cm^–1^ resolution and averaged over 500 acquisitions on a Bruker Vertex V70 spectrometer using a Specac golden gate ATR accessory. Spectral deconvolution was achieved using multipeak fitting software (OriginPro), and the band centers were determined using a second-derivative peak-finding analysis developed in house.

GC–MS was carried out using a Varian CP3800 GC–MS equipped with a Varian Saturn mass spectrometer detector (MSD) and a 30 m × 0.25 mm i.d, 0.25 μm film thickness CD-5 (ChromatographyDirect.com) capillary column. It is coated with poly(5% diphenyl/95% dimethylsiloxane) and can withstand temperatures of 325/350 °C. This column is suitable for boiling-point elution ordering and is slightly more selective for aromatic compounds. Samples for analyses were dissolved in n-hexane and 1 μL injected on-column with helium as a carrier gas. A split ratio of 15:1 was used. A typical mass selective detector ionization energy of 70 eV, an emission current of 10 μA, and a scan time of 1 s/scan were used to detect mass-to-charge ratios (*m*/*z*) between 40 and 450.

Samples for NMR were dissolved in deuterated chloroform (CDCl_3_) and recorded on a Bruker 500 MHz instrument. For proton NMR, conditions were: width 10,000 Hz (−4.01 to 15.97 ppm), 5° pulse, dwell time 50 μs, digital resolution 0.3 Hz/point. 32 scans were averaged, and the acquisition time was 3.27 min. For carbon NMR: width 34722 Hz (−27.9 to 248.1 ppm), 30° flip angle with power gated decoupling, dwell time 50 μs, digital resolution: 0.33 Hz/point. 512 scans were averaged, and the acquisition time was 3.02 min. The two-dimensional (2D)-QF-COSY data shown in Supporting Information, Figures S4 and S5 were recorded using a 700 MHz Avance II+ Bruker spectrometer, with nonuniform sampling, spectral width of 9090 Hz (12.98 ppm), 110 *t*_1_ increments, a constant time *t*_0_ of 0.22 s. Processing was performed in TopSpin 4.0.9 software.

## 3 Results and Discussion

### 3.1 Refractive Index Measurements

**Figure 3:**
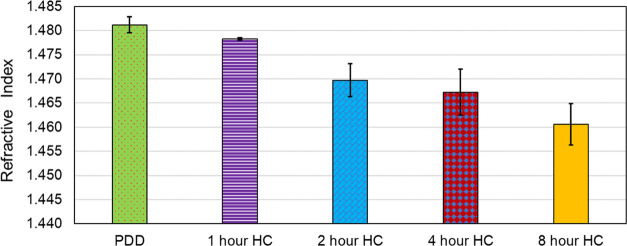
Refractive index of the samples at 20 °C.

Refractive index (RI) measurement is a simple method of assessing the degree of change in a sample post hydrogenation. Figure 3 shows the average refractive index of samples recorded at different stages of hydrogenation. The initial refractive index of phenyldodecane is 1.480 ± 0.0005 (PDD), which gradually reduces to 1.461 ± 0.004 after 8 h of hydrogenation. Generally, aromatic molecules have a higher refractive index; hence, the reduction in refractive index upon hydrogenation suggests that a proportion of PDD itself has decreased due to hydrogenation. The largest decrease in refractive index occurs in the first 2 h of hydrogenation.

### 3.2 UV–Vis Spectroscopy

**Figure 4:**
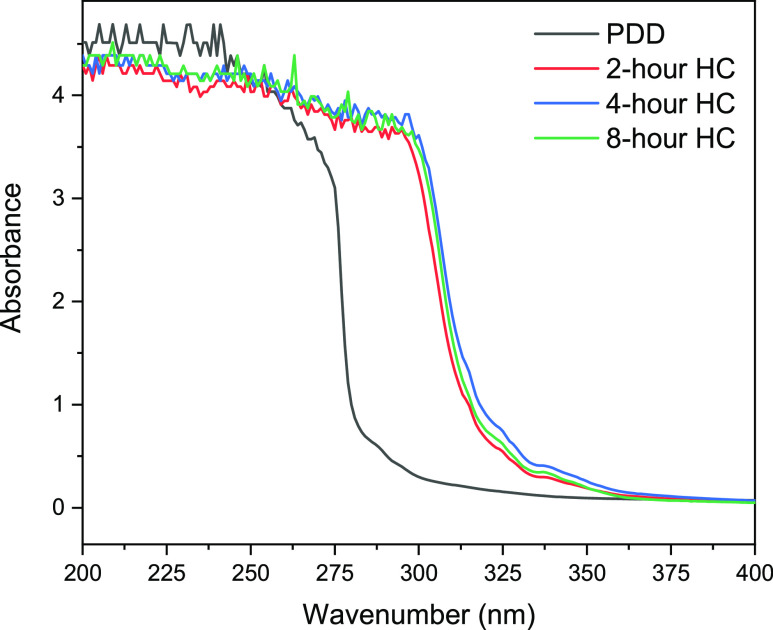
UV–vis spectra of the samples in the range of 200–400 nm.

The UV–vis spectra presented in Figure 4 show that the hydrogenation of PDD produces chemical changes in the sample because the absorbance between 200 and 250 nm is reduced while that between 275 and 375 nm increases. The broad absorption band between 200 and 300 nm shows that monoaromatic molecules are still present in the hydrogenated samples. Molecules responsible for absorption in the 260–350 nm region are usually conjugated molecules, polyaromatic, polycyclic, or naphthenic hydrocarbons.^10^ Polyaromatic molecules (such as naphthalene, anthracene, phenanthrene, and pyrene) with no nitrogen or sulfur atom substitution will generally have a higher refractive index than monoaromatic compound, while the results of refractive index shown in Figure 3 show that the RI is in a decreasing trend. Therefore, it is safe to infer that the products responsible for absorption in the 260–350 nm region are either conjugated molecules or monocycloalkane. Conjugation such as in dienes and trienes generally results in bathochromic and hyperchromic shifts in absorption, which explains the increased intensity in the region. However, further interpretation of these bands based on their intensity is impractical as this technique is sensitive to the degree of conjugation. Thus, UV–vis spectroscopy while only suitable as a quality control technique does nevertheless give the intensity at a chosen wavelength for comparison with a standard value.

### 3.3 GC–MS

**Figure 5:**
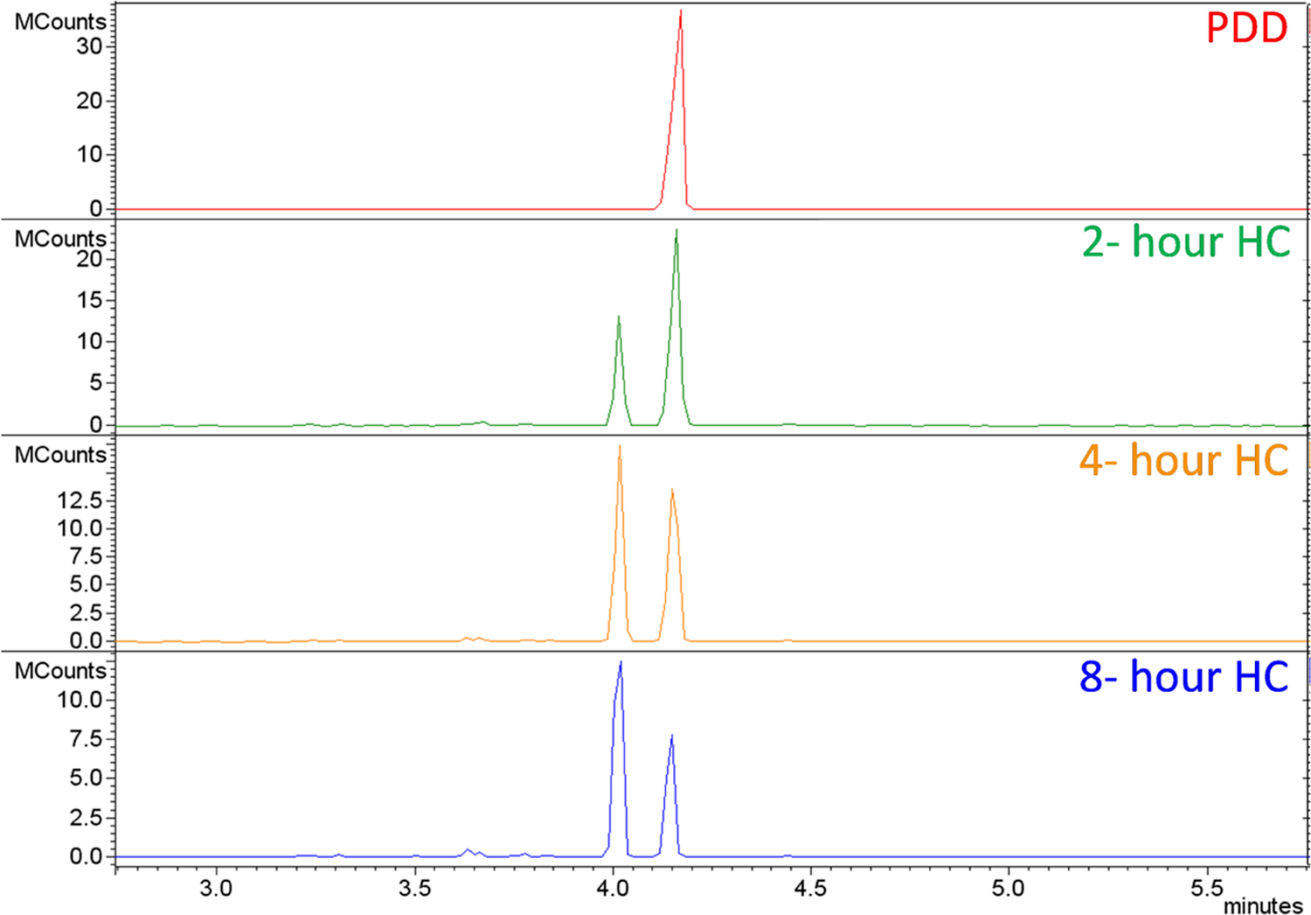
Gas chromatography of PDD and its hydrogenated samples between 2.5- and 6.0-min retention times.

**Figure 6:**
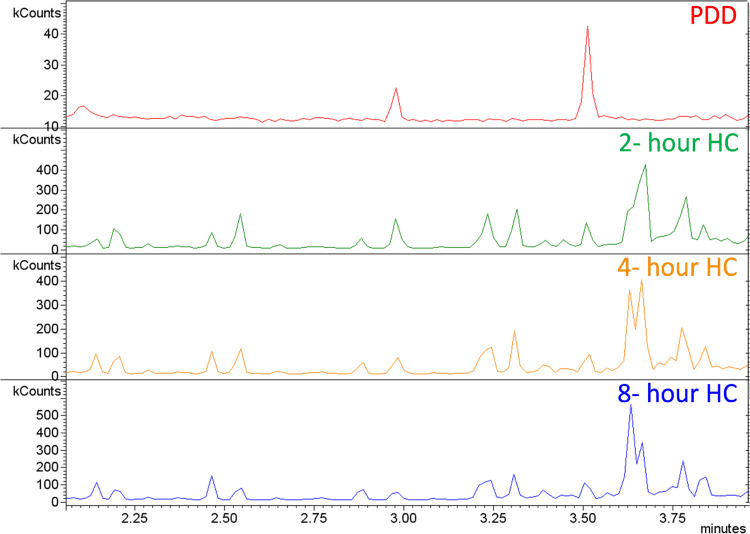
Gas chromatography of PDD and its hydrogenated samples between 2- and 4-min retention times.

Figure 5 shows the chromatogram of samples collected after 3 different hydrogenation time periods under the conditions mentioned in the Materials and Methods section. The sharp peak at 4.15 min is due to phenyldodecane itself. The formation of new molecules is demonstrated by the presence of a new peak at the retention time of 4 min. When using a CD5 nonpolar column, the retention time increases with an increase in boiling point. This suggests that the new molecule formed is of lower boiling point. Furthermore, the yield of this new molecule increases with hydrogenation time while the peak from PDD decreases. Examination of the *m*/*z* ratios and library match suggests the formation of cyclohexyldodecane, the expected product of hydrogenation of the benzene ring of PDD to cyclohexane. However, hydrocarbon chain scission may also occur, which would generate shorter-chain-length molecules which would be expected to elute at shorter times. Evidence for these was found in the chromatograms between 2- and 4-min elution times, Figure 6. A library match suggests the formation of cyclohexyl-octane or cyclohexyl-hexane indicating a decrease in the length of the aliphatic side chain. Peak assignments were made by matching to mass spectral libraries such as NIST and Wiley, as shown in Table S1. This is clear evidence that catalytic thermal cracking of the aliphatic chain occurs during hydrogenation. It is concluded that hydrogenation in the presence of the Pt on alumina catalyst at high temperatures and pressures, i.e., our experimental condition, involves both the saturation of the aromatic ring via hydrogen addition and the catalytic cracking of the hydrocarbon chain into smaller chains, including volatile gaseous molecules which are lost when the reactor is opened to retrieve samples.

### 3.4 FTIR Spectroscopy

**Figure 7:**
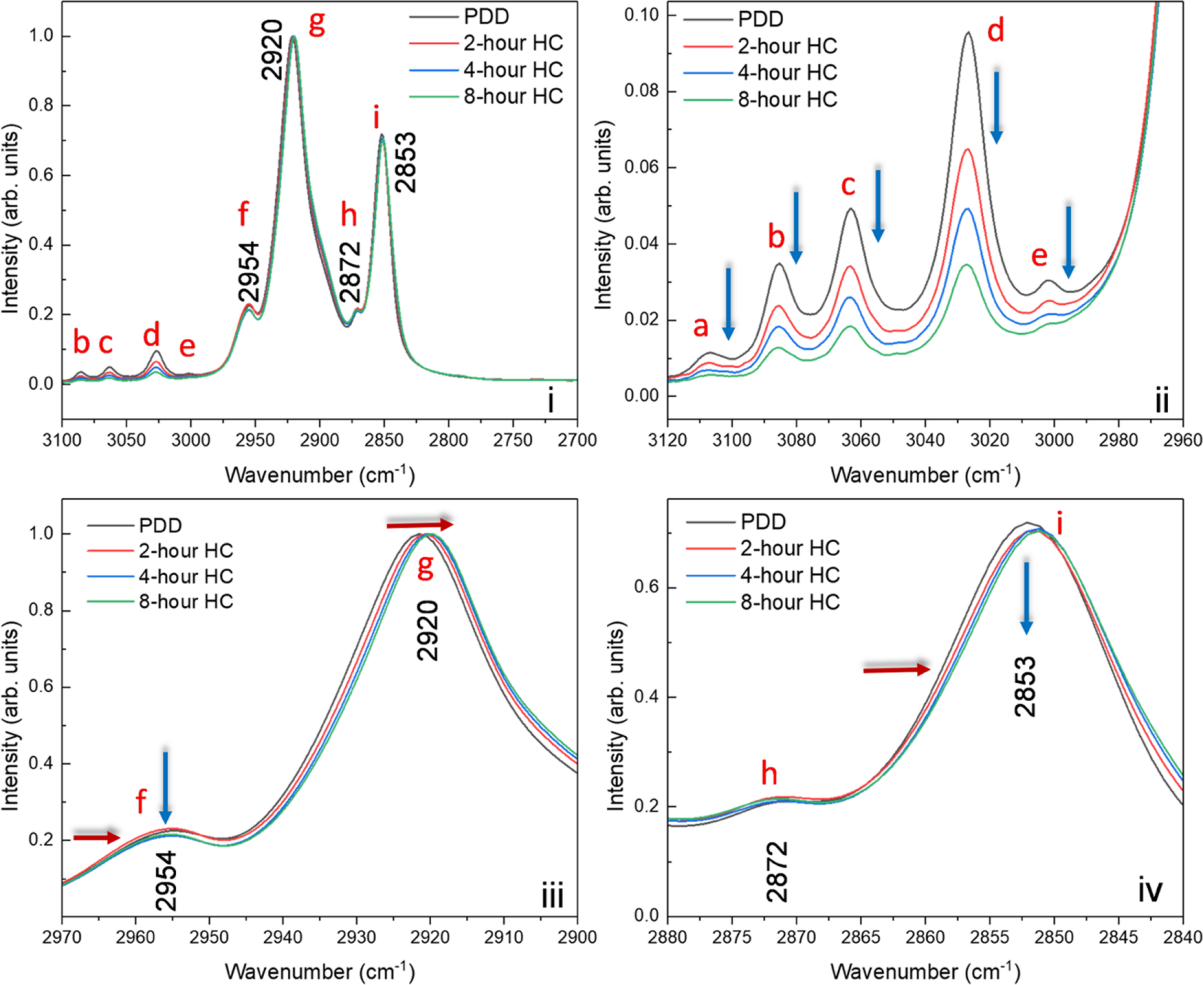
FTIR spectra of the samples in the ranges (i) 3100–2700 cm^–1^, (ii) 3120–2960 cm^–1^, (iii) 2970–2900 cm^–1^, and (iv) 2880–2840 cm^–1^. The normalization [0,1] is carried out in the region 3400–2700 cm^–1^.

**Figure 8:**
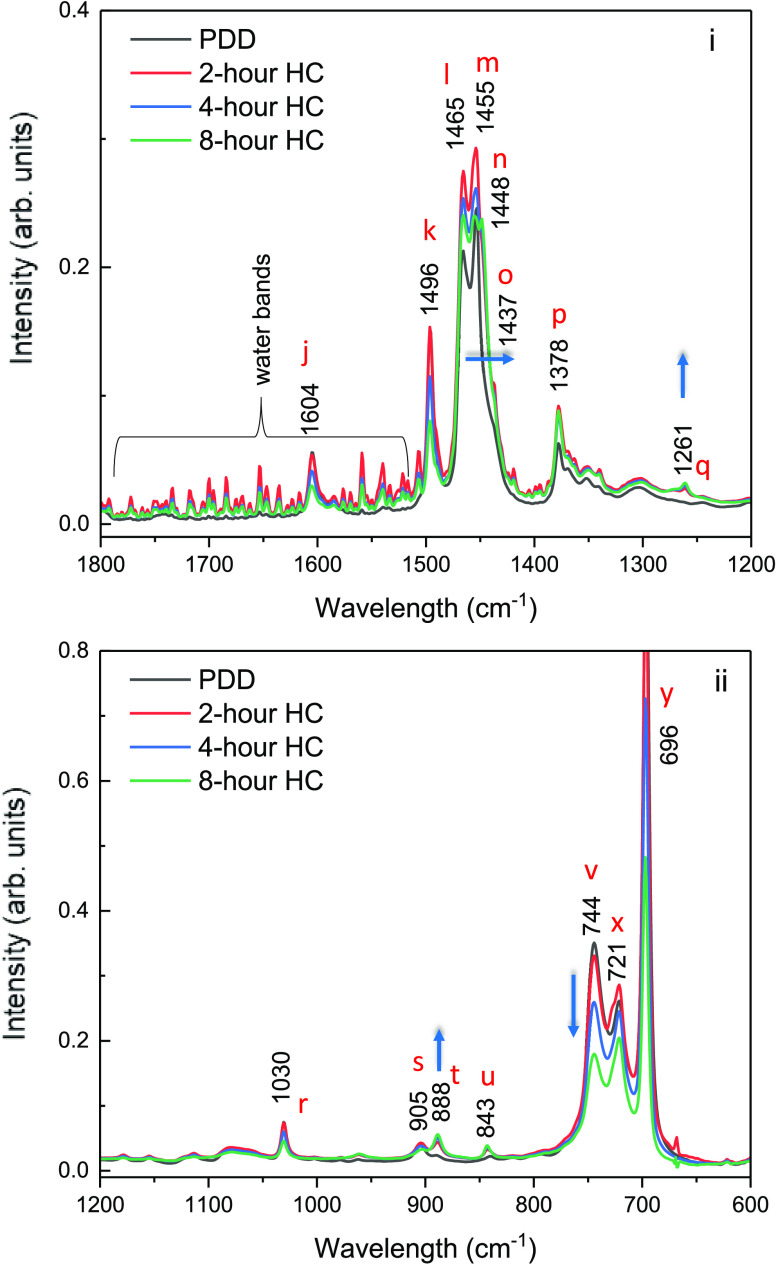
FTIR spectra as PDD is hydrogenated, in the ranges (i) 1200–1800 cm^–1^ and (ii) 600–1200 cm^–1^. For clarity, band shifting in frequency is indicated by the horizontal arrow and in intensity by vertical arrows.

The PDD infrared spectra of interest lie in the region between 2700 and 3100 cm^–1^, as shown in Figure 7, and in the region between 1800 and 600 cm^–1^, as shown in Figure 8. The band assignments required for analyzing these spectra are taken from the literature and given in Table 2. In the C–H stretching region (Figure 7), the survey spectrum is shown in panel (i) and expanded spectra in panels (ii) to (iv). The peaks at 2872 cm^–1^, 2890 (shoulder) cm^–1^, and 2954 cm^–1^ can be assigned to the symmetric stretch (*r*^+^), Fermi Resonance band (*r*_FR_^+^), and asymmetric (*r*^–^) stretching modes of the methyl groups in the chain. The much stronger bands at 2921 and 2853 cm^–1^ are assigned to the corresponding symmetric (d^+^) and asymmetric (d^–^) C–H stretching modes of the methylene groups. Their much higher intensity compared to the methyl modes points to the presence of linear chains with many methylene groups and fewer side chains containing methyl groups. The methylene bands at 2921 and 2853 cm^–1^ at various stages of hydrocracking, see Figure 7iii,iv, show a shift to lower frequency at longer hydrogenation times. To emphasize the changes in their intensity, the spectra are normalized to the strongest band at 2920 cm^–1^.

**Table 2:** FTIR Band Assignments and Positions^a^

peak position (cm^–1^)	assignment	literature value (cm^–1^)	ref	significance
Aryl C–H Stretching Vibration (3100–3000 cm^–1^)				
3106 (w)	nonterminal C–H stretch in propene substructure	3104 ± 7	(11)	4 bands associated with mono-substituted aromatic ring. Their intensity decreases with hydrogenation.
3026 (w)	out-of-plane methyl stretch in propene substructure	3036 ± 12	(11)	
3063 (w)	out of plane methyl stretch in cis-2-butene substructure	3060 ± 4	(11)	
3085 (w)	CH stretch in vinyl group	3080	(12)	
Alkyl C–H Stretching Vibration (3000–2850 cm^–1^)				
2954 (w)	CH_3_ asymmetric C–H stretching vibration, *r*^+^	2953	(13)	subtle decrease in intensity and shift to lower wavenumbers
2921–2916 (w)	CH_2_ asymmetric C–H stretching vibration, d^–^	2920	(13)	shift to lower wavenumbers
2890–2894 (sh)	CH_2_ Fermi resonance, *r*_FR_^+^	2890	(13)	shift to lower wavenumbers
2872 (s)	CH_3_ symmetric C–H stretching vibration, *r*^+^	2873	(13)	
2853 (s)	CH_2_ symmetric C–H stretching vibration, d^+^	2850	(13)	subtle decrease in intensity and shift to lower wavenumbers
1496 (s)	aromatic C=C stretch	1495	(12, 14)	intensity decreases
1465 (s)	H–C–H bend	1467	(13, 14)	intensity first increases then decreases
1455 (s)	alkyl CH scissoring	1454	(12, 14)	intensity first increases then decreases
1448 (w, sh)	CH_2_ scissoring	1449	(12, 14)	intensity first increases then decreases
1437 (sh)	unsubstituted methylene group adjacent to a double bond (polypropylene)	1436	(14−16)	intensity first increases then decreases
Fingerprint Region (1500–600 cm^–1^)				
1387 (sh)	isopropyl CH_3_ CH bending in a branched paraffin substructure	1385	(12)	intensity first increases then decreases
1378 (s)	CH_3_ symmetric CH bending in an unbranched paraffin substructure	1378	(12)	intensity first increases then decreases
1368 (sh)	isopropyl CH_3_ CH bending in a branched paraffin substructure	1367	(12)	intensity first increases then decreases
1363 (sh)	tertiary butyl CH_3_, CH bending in a branched paraffin substructure	1365	(12)	intensity first increases then decreases
1350 and 1340 (sh)	CH_2_ wagging in the paraffin substructure		(17)	intensity first increases then decreases
1261 (w) and 1244 (vw)	CH_2_ twisting in the paraffin substructure		(17)	intensity increases
1178, 1165, 1166, 1145, and 1030 (vw)	C–C stretching modes of mono-substituted aromatic		(17)	intensity decreases
905 (s)	=CH_2_ wag	908	(12)	intensity decreases
888 (s)	C=CH_2_ out of plane deformation in vinylidene substructure	888	(12)	intensity increases
843 (w)	aromatic CH out of plane deformation from pyrene-like substructure	843	(18)	intensity decreases
744 (s)	aromatic C–H out-of-plane deformation	774.5	(12)	intensity decreases
721 (s)	CH_2_ rocking vibration	720	(13)	intensity decreases
696 (s)	aromatic CH out of plane deformation in alkyl benzene substructure	698	(12)	intensity decreases

a s, strong; w, weak; sh, shoulder; v, very.

Figure 7iv shows similar very small changes in intensity and a shift to lower wavenumbers for the infrared bands at 2872 cm^–1^ (CH_3_ sym str.) and 2853 cm^–1^ (CH_2_ sym str.), respectively. The near-constant intensity of the aliphatic stretching bands after hydrocracking is to be expected because of hydrocracking, and the original chain now becomes more aliphatic chains. The presence of a higher proportion of smaller molecules can also cause the shift to lower wavenumbers which corroborates with the GC data shown in Figure 6. In contrast, the aromatic bands between 3000 and 3120 cm^–1^ (Figure 7ii) show a substantial decrease in their relative intensity with the duration of hydrocracking providing unambiguous evidence that the aromatic ring is being hydrogenated.

Changes in the intensity ratio of the asymmetric methylene to asymmetric methyl stretching bands is a pointer toward the degree of branching and chain length. An increase in the ratio of the intensities would suggest saturation of the aromatic ring, while a decrease would suggest shorter chains. A lower CH_2_/CH_3_ ratio could result, for example, from branching (isomerization) or ring opening, leading to a greater number of methyl groups.

The intensity ratios of the CH_2_ to the CH_3_ asymmetric bands are given in Table 3 and show that after increasing up to 4 h, they have decreased after 8 h of hydrocracking, suggesting saturation of the aromatic carbons via hydrogen addition followed by further hydrogen addition leading to opening of the ring resulting in a branched molecule. Thus, a change in reaction mechanism occurs. Hydrogenation of aromatic compounds is a reversible process under the conditions used here, and both the reactant (PDD) and the products of hydrogenation reach an equilibrium in the sample.^19^ This makes quantitative interpretation of spectroscopy measurements difficult. Moreover, there is a decline in the yield at each subsequent hydrogenation stage. It can be concluded that if hydrogenation in the presence of a 0.5% Pt on alumina catalyst is carried out indefinitely, the sample will eventually be converted to volatile gases composed of short-chain hydrocarbons.

**Table 3:** Ratio of the Intensities of the Asymmetric Methylene to Asymmetric Methyl Stretching Bands

sample name	asymCH_2_/CH_3_
PDD	4.4_4_
2 h hydrocracked	4.3_3_
4 h hydrocracked	4.7_1_
8 h hydrocracked	4.6_3_

The hydrogenation of a monoaromatic molecule like PDD in the presence of 0.5% Pt on alumina catalyst produces a wide range of products. There are many possibilities for symmetric and asymmetric hydrogenation of the aromatic ring leading to complete or partially hydrogenated intermediates.^20^ Also produced are the products of direct thermal cracking. Attack at the carbon ipso position followed by hydrogen addition leads to the formation of olefinic molecules, normal paraffins, and gaseous hydrocarbons.^21^ This means that the average alkyl chain becomes shorter, and there is then a reduced yield of saturated naphthenic hydrocarbons. The spectra of these molecules mainly occur in the lower-wavenumber IR regions and are shown in Figure 8i,ii. Specifically, Figure 8i (1800–1200 cm^–1^) shows the conjugated C=C stretching band at 1604 cm^–1^ (denoted as j in Figure 8i). This decreases with hydrocracking time, which confirms the effectiveness of the catalyst in hydrogenating the aromatic ring. However, even after hydrocracking for 8 h, some aromatic molecules remain, which shows that hydrogenation of monoaromatic molecules is to some extent an equilibrium process as mentioned earlier.

Additionally, the intensity of bands assigned to aromatic C=C stretches (see peaks denoted “k” in Figure 8i and Table 2) decreases monotonically with hydrocracking time, while peaks associated with methyl, methylene bend, and scissoring (see peaks denoted l-p in Figure 8i and Table 2) all appear to first increase in intensity after 2 h of hydrocracking, followed by a decrease with hydrocracking time, suggesting a reaction mechanism that involves the formation of stable reaction intermediates. These have undergone partial hydrogen addition and then undergo further conversion to form a saturated monocyclic molecule, and hence there is a decrease in the intensity of these bands after first increasing. The shoulder band positioned at 1437 cm^–1^ is assignable to an unsubstituted methylene group adjacent to a double bond or to a conjugated ring (such as cyclohexadiene or cyclohexene) and points to the formation of an intermediate with unsaturation. The increase in intensity of the CH_3_ symmetric rocking mode vibration at 1378 cm^–1^ (peaks denoted p in Figure 8i) and the CH_2_ twisting modes of a paraffin observed at 1261 cm^–1^ (peaks denoted q in Figure 8i) suggest the formation of branched and unsymmetric structures. Such molecules are formed due to ring opening or paraffin chain scission. Generally, ring-opening reactions occur at lower temperatures;^22^ therefore, it is concluded that the ring-opening reaction mechanism occurred during the temperature-increasing step as the reactor was heated to 400 °C from room temperature.

Figure 8ii shows FTIR spectra in the 600–1200 cm^–1^ region. Bands centered at 1030 cm^–1^ and at 905, 744, 721, and 696 cm^–1^ (peaks denoted *r*–*y* in Figure 8ii and Table 2) can clearly be seen to decrease with hydrocracking time, while bands at 888 and 843 cm^–1^ (peaks denoted t and u in Figure 8ii) increase in intensity. The increase in intensity is due to the presence of partially saturated intermediates. An interesting feature post hydrocracking is the change in the relative intensity of *I*_888_/*I*_905_ and *I*_744_/*I*_721_ readily seen in Figure 8ii. The increase in the *I*_888_/*I*_905_ ratio suggests the formation of shorter paraffinic chains, while the decrease in the *I*_744_/*I*_721_ ratio suggests a reduction of aromatic content post hydrocracking.^23^ Therefore, the spectral features described here, and their changes, point to changes in the structure of the PDD molecule due to saturation of the aromatic ring by hydrogen addition, changes in the substitution via ring opening and paraffin chain scission, and finally the composition (by virtue of it being a mixture of unreacted sample, final products, as well as stable intermediates).

### 3.5 Nuclear Magnetic Resonance Spectroscopy

NMR spectra further confirm that PDD had been hydrogenated qualitatively following the decrease in the aromatic chemical shifts and the considerable increase in those from new products. From the GC and FTIR spectra, it is established that the sample obtained post hydrogenation is a mixture of various closely associated molecular structures. Sarpal et al.^24^ and Mäkelä and co-workers^25^ have developed methods of using ^13^C NMR chemical shifts to identify specific carbon structures in a mixture such as mineral oil. The earlier work of Sarpal et al. used one- and two-dimensional NMR to specifically identify normal and branched paraffin structures in selected base oils.^26^ (Their assignments are given in their Table 2.) We have used the results from both groups to support the assignments proposed below.

#### 3.5.1 ^1^H NMR

**Figure 9:**
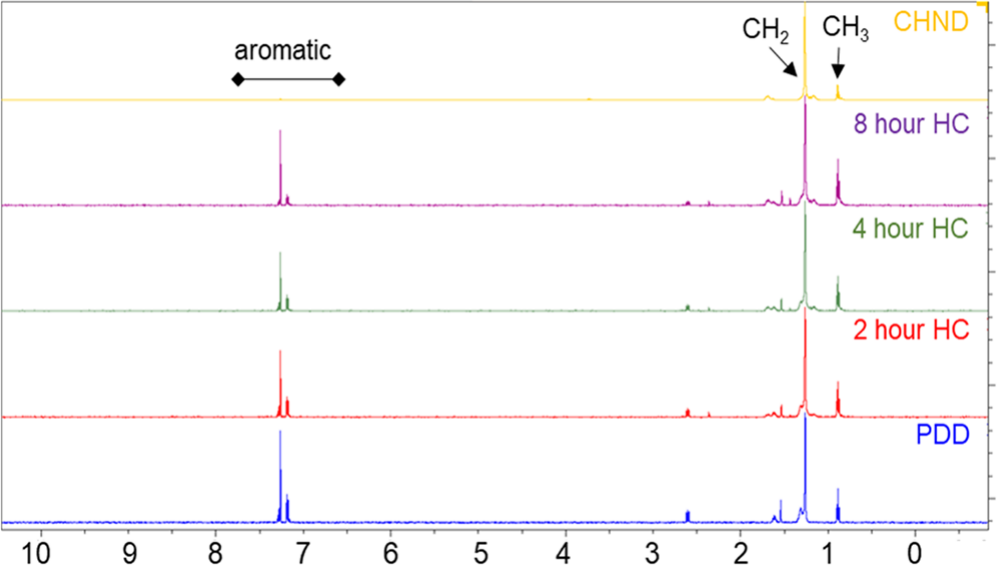
^1^H proton NMR chemical shifts of the samples.

The survey proton NMR spectra of all samples pre and post hydrogenation are shown in Figure 9 after normalization to the methylene peak at 1.26 ppm, which is from the proton of the branchless methylene group (BL, see Figure 1). The singlet signal at 7.26 ppm is from the chloroform (CDCl_3_) solvent. The spectra show the presence of aliphatic peaks from methyl and methylene (CH_3_ and CH_2_) groups characteristic of long-chain alkanes as well as from aromatic features. In contrast, the proton NMR spectra of CHND overlaid on the figure shows no aromatic peaks.

**Figure 10:**
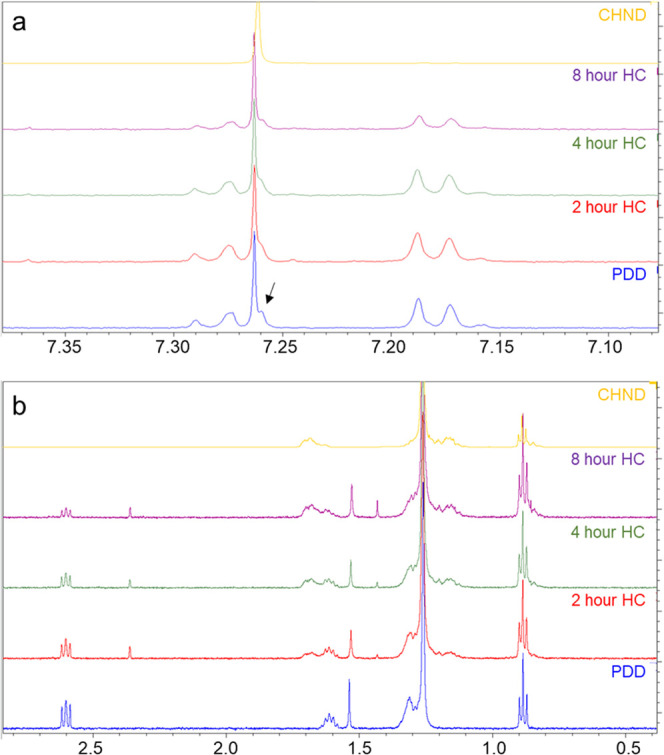
Proton NMR spectra of all samples in the (a) aromatic and (b) aliphatic regions.

Figure 10a shows details of the aromatic region where the peaks between 7.15 and 7.20 ppm are attributed to protons attached to carbon at the para position and between 7.25 and 7.30 ppm to protons attached to carbon at ortho and meta positions. The decrease in the intensity of these peaks with hydrogenation time, falling to approximately 50% of their initial value, is direct evidence of the conversion of aromatic molecules to other species. The shoulder feature indicated by the arrow in Figure 10a also decreases gradually on further hydrogenation.

Figure 10b shows details of the aliphatic region in ^1^H NMR. The triplet of peaks around 2.6 ppm is due to protons of methylene groups at the α position of PDD (see Figure 1), and these decrease with longer periods of hydrogenation, evidencing changes in the neighboring proton environment. Generally, the peaks around 1.3–1.72 ppm are characteristic of protons of the methylene groups in cyclohexane and so are observed in CHND. In the hydrogenated samples of PDD, their relative intensity increases with time, which points to the generation of increased quantities of saturated ring molecules in the sample. The two overlapping peaks between 1.65 and 1.7 ppm are from protons at the ortho and para positions of cyclohexane and their intensity increases. The two peaks between 1.5 and 1.65 ppm are due to protons at the β position (see Figure 1), and in contrast, their intensity gradually decreases with hydrogenation time chiming with the increase in saturation by hydrogen addition. The single peak at 1.54 ppm gradually shifts to lower ppm while its intensity with respect to the CH_3_ singlet at 1.26 ppm first decreases and then slightly increases with hydrocracking time. A strong coupling effect between the protons can cause distortion of the peaks and cause small chemical shifts.

**Figure 11:**
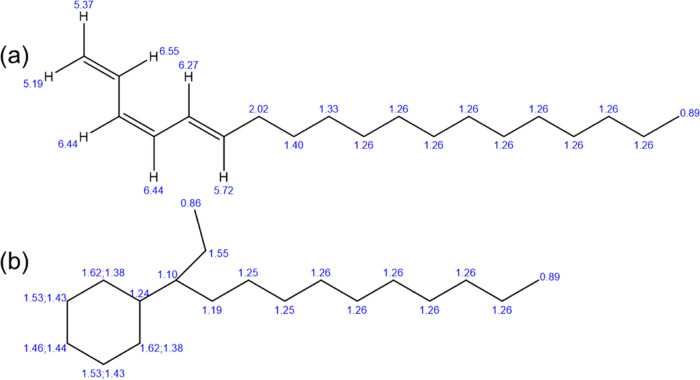
Proposed product structures: (a) conjugate structure combined with linear chain (b) ring with chain branching at α position. ^1^H NMR shifts at each C calculated using ChemDraw Professional.

To aid in assigning the species giving rise to new spectra, which are often of low intensity, shifts were calculated for conjugated and cyclohexane ring structures with ring branching using ChemDraw. These structures and the associated shifts at specific carbon atoms are shown in Figure 11.

A peak at 1.43 ppm increases with hydrocracking but is not observed in either PDD or CHND. This is evidence of conjugation that occurs in dienes and trienes, but as implied in Figure 11a, it is at least two carbon atoms away. The intensity of this peak increases with hydrogenation time, which suggests opening of the benzene ring to produce conjugation. The presence of a peak between 1.3 and 1.35 ppm suggests substitution at α or β positions implying the presence of 2-phenyldodecane and 3-phenyldodecane as impurities in 1-phenyldodecane. Its intensity reduces with hydrogenation, implying that isomers are also hydrogenated in a similar manner to PDD itself. The peaks between 1.1 and 1.2 ppm are absent in PDD but present in hydrogenated samples and in CHND. These are from the protons of the methylene groups at the α position (see Figure 11b). A triplet at 0.89 ppm is from the methyl protons at the ends of the aliphatic chains of PDD and CHND, and as anticipated, its intensity does not change with hydrogenation time. However, smaller new satellite peaks, up field of the 0.89 ppm feature, at 0.85 and 0.84 ppm appear after 4 and 8 h, suggesting an increase in methyl or ethyl branching at α-positions (see Figure 11b).

The change in methyl branching that can occur on hydrocracking is of particular interest and has been mentioned earlier in the IR analysis. It has been pointed out by Sarpal and co-workers that spectra in the region between 10.0 and 23.0 ppm indicate the presence of different branched structures. They showed that cross-peaks in the COSY spectrum arise from coupling of C–H and CH_3_ groups and CH and CH_2_ groups in hydrotreated base stocks. Furthermore, they used 2D HETCOR to arrive at the ^13^C shifts for normal and branched paraffin structures. These have been used to aid the assignments in this work as presented below. Figure S5 in the Supporting Information shows the ^1^H COSY spectra recorded for PDD before and after hydrogenation showing changes analogous to those reported for oil by Sarpal et al. The COSY spectra in Figure S6 show the close similarity of the hydrogenated PDD to the spectra of CHND.

#### 3.5.2 ^13^C NMR

**Figure 12:**
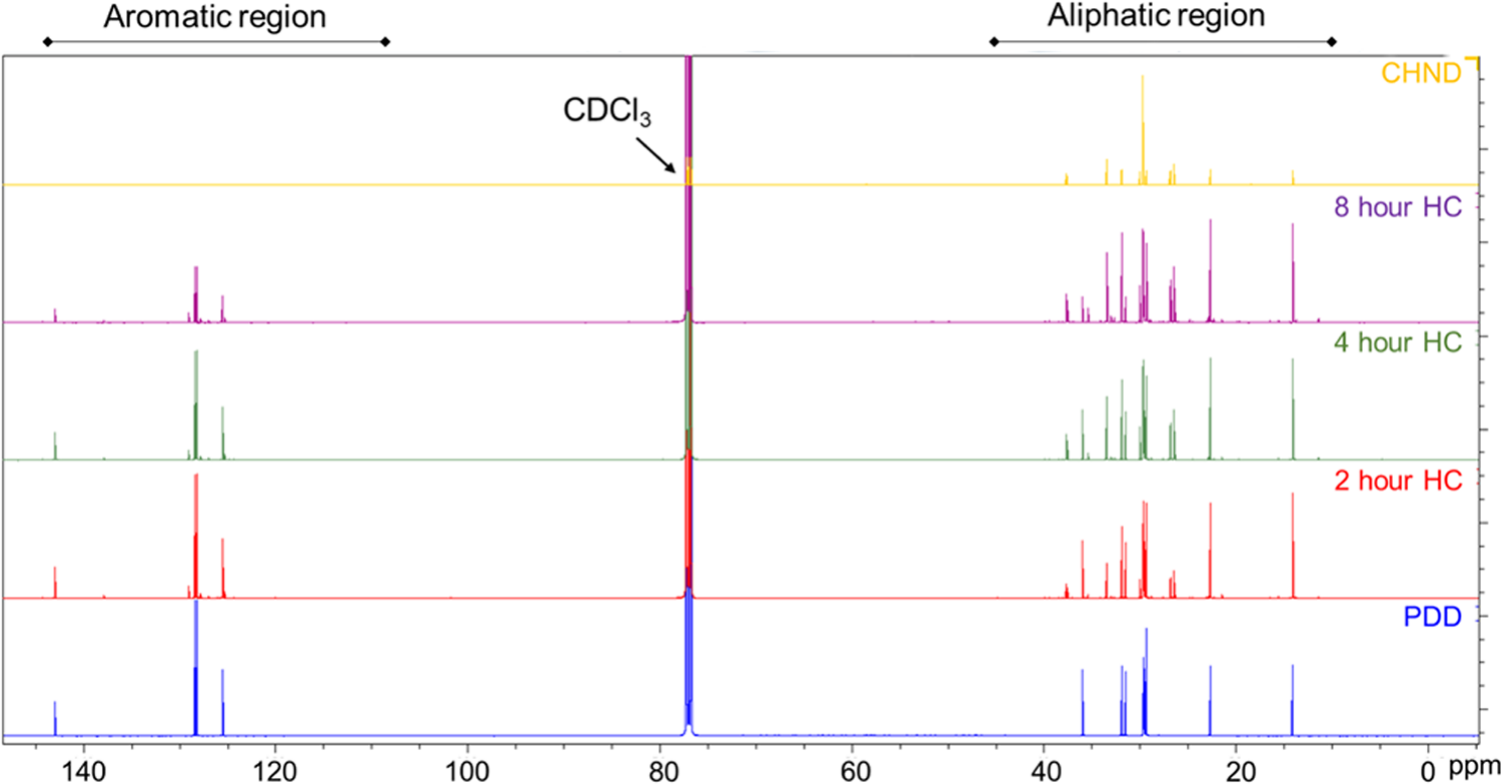
^13^C NMR survey spectra of the hydrogenated samples.

**Figure 13:**
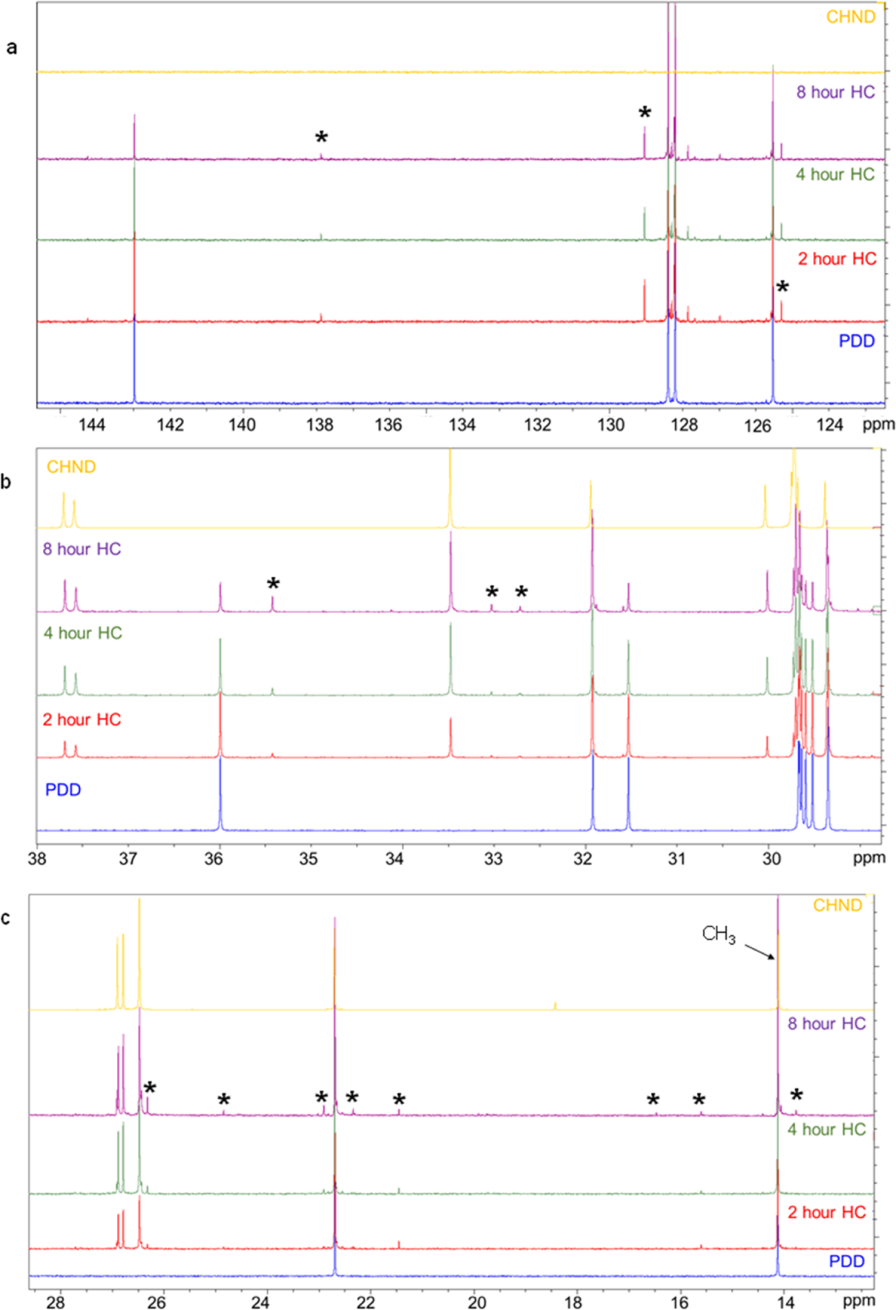
Detailed ^13^C NMR spectra of the samples in the (a) aromatic region and (b, c) aliphatic region, showing the presence of new species and changes in signal relative intensity. Peaks absent in both PDD and CHND samples but present in hydrogenated samples are denoted by the * symbol.

The ^13^C NMR survey spectra of the samples are shown in Figure 12. The aliphatic and aromatic carbon shifts fall in the ranges of 10–50 and 90–150 ppm respectively. (The chloroform solvent (CDCl_3_) gives a triplet of peaks between ∼77.0 and 77.5 ppm.) A general comparison with the PDD spectra in the aliphatic region shows new peaks in the hydrogenated samples, and these are indicated by ∗ in the detailed spectra in Figure 13a–c (aromatic region, Figure 13a: aliphatic region, Figure 13b,c).

In the aromatic region (Figure 13a), peaks related to monoaromatic PDD gradually decrease with hydrogenation time. These peaks are related to the carbons in the benzene ring, and details are given in Table 4. Peaks in the aliphatic region are shown in Figure 13b,c. Table 5 shows a list of peaks that increase with hydrogenation time. These peaks are related to carbon atoms of a monocycloalkane such as CHND. In addition to the ^13^C NMR peaks that are associated with either PDD or CHND molecules, new peaks are observed in the hydrogenated samples which are shown in Table 6. Supporting Information Figures S1–S4 are provided to aid in high-resolution inspection and quantifying the changes in peak intensities and shifts.

**Table 4:** ^13^C NMR Peaks That Decrease in Intensity with Hydrogenation Time and Their Assignment and Structural Significance

peak position (ppm)	assignment	significance
142.96	carbon at the ipso position of the benzene ring	reduced presence of aromatic ring structure
128.38	carbon at meta position of the benzene ring	
128.19	carbon at ortho position of the benzene ring	
125.5	carbon at para position of the benzene ring	
35.98	methylene at the α position to the benzene ring	
31.528	methylene carbon at the β position	
29.34	methylene carbon at γ position in PDD	
29.51, 29.59, 29.63, 29.67	branchless carbons in the aliphatic chain (BL)	shorter branchless chain

**Table 5:** ^13^C NMR Peaks That Increase in Intensity with Hydrogenation Time and Their Assignment and Structural Significance

peak position (ppm)	assignment	significance
37.68	methine node of cyclohexane	increased presence of saturated ring structure
37.57	methylene carbon at the α position to the cyclohexane ring	
33.47	methylene carbon of monosaturated alkane at the ortho position	
29.36	carbon at γ position of CHND	
26.45	methylene carbon at meta position of CHND	
29.65, 29.69, and 29.72	BL carbons with minor changes in the neighboring carbon bond adjacent to α and β positions	suggests formation of new species via ring opening and formation of ethyl or branching
26.89	26.89 is split into peaks at 26.9 and 26.88	
31.924	methylene carbon at the S3 position of aliphatic chain	

**Table 6:** New ^13^C NMR Peaks Only Observed in Hydrogenated Samples and Their Assignment

new peaks (ppm)	assignment	significance
137.87	carbon in a conjugated substructure such as dienes and trienes	decreasing intensity suggests stable reaction intermediates
129.03	carbon at meta position affected by change in the environment of neighboring carbons	
125.56	carbon at ortho position affected by neighboring carbons	
125.29	carbon at para position affected by neighboring carbons	
128.10, 127.97, 127.84, 127.65, and 126.97	conjugated molecular substructures with no correlation between the hydrogenation time and its intensity	new stable intermediate species
125.29, 128.33, 128.29, 128.25, and 128.22	conjugated molecular substructures that increase in intensity in the first 2 h of hydrogenation and then decrease	new stable reaction intermediates
125.7	doublet formed after 2 h of hydrogenation, its relative intensity changing after 4 and 8 h of hydrogenation.	increasing intensity suggests stable products via ring opening and branching
35.42, 34.12, 33.02, 32.71	new stable intermediate species	decreasing intensity suggests stable reaction intermediates
30.01, 26.32 24.84, and 22.90, 22.54, 22.33, and 21.45 (with satellites at 22.36 and 22.30)	ethyl branch in the vicinity of the BL structure	increasing intensity suggests stable products via ring opening and branching
26.47, 26.45, and 26.43	ethyl or methyl branching in the neighborhood of a methylene carbon at the β-position	
22.82 and 22.65	satellite peaks present in hydrogenated samples as well as in CHND	
16.46, 15.6, 14.05, 13.83, and 13.76	new stable intermediate species with no correlation between their hydrogenation time and their intensity	new stable intermediate species
11.46	methyl carbon at position S1 when there is a methyl or ethyl branch at the S3 position	increasing intensity suggests ring opening resulting in some iso-paraffinic substructure
14.11	terminal methyl carbon at the S1 position	first increases and then decreases with hydrogenation period

**Figure 14:**
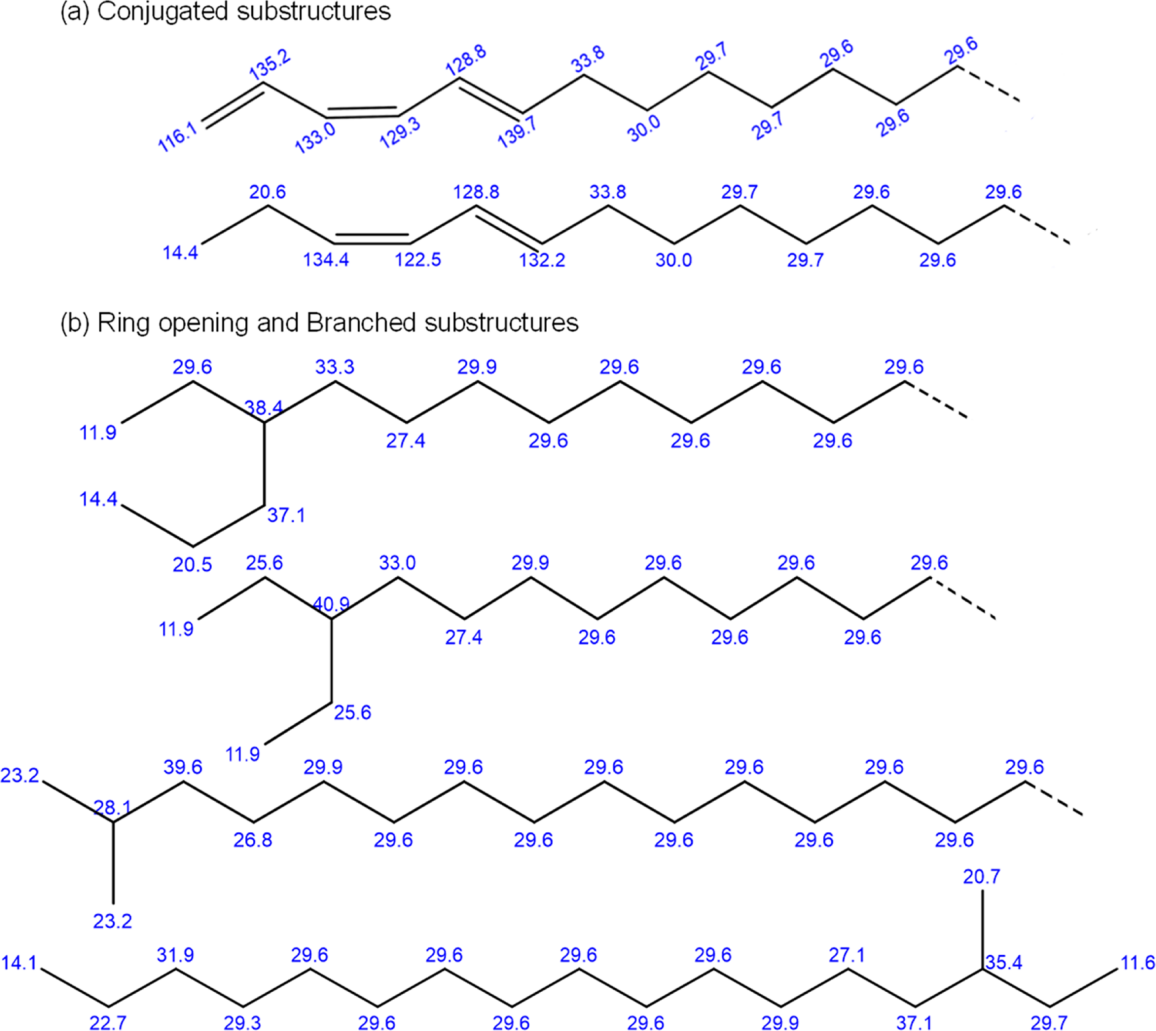
Likely substructures: (a) conjugated and (b) ring opening and branched, formed on hydrogenation and their NMR positions given by ChemDraw.

The presence of new peaks suggests the formation of new species due to the hydrogenation of the monoaromatic compound. To aid in the assignment of these peaks, shifts associated with several potential product molecules were calculated using ChemDraw, which are shown in Figure 14. Although the conversion of a monoaromatic compound to a saturated monocycloalkane occurs, new and different byproducts are also formed. Some species are stable byproducts, while others such as the species containing conjugated substructures are intermediate stable molecules that subsequently get saturated. All of those peaks in Table 6 that increase after 2 h and then gradually decrease imply the occurrence of different catalytic reaction products and pathways. Hence, these results corroborate the results obtained from GC–MS and FTIR. NMR, however, helps to provide greater details of the molecular structures that can be produced post hydrogenation.

## 4 Conclusions

A high-pressure and high-temperature benchtop mini-reactor has been designed and used to carry out hydrogenation of phenyldodecane (PDD)—a model monoaromatic compound with a long linear aliphatic tail, in the presence of a 0.5% platinum on alumina catalysts. Refractive index measurements were used to quantitatively assess the changes that occur with hydrocracking time, while powerful complementary spectroscopic tools were used to assess the corresponding chemical changes. UV–vis spectroscopy, which is intrinsically sensitive to aromatic compounds, suggests that only a fraction of the aromatic compounds were converted to either conjugated molecules or mono-cycloalkanes, indicated by the absorption intensity between 260 and 350 nm increasing. GC–MS studies showed that in the hydrogenation process, saturation of the aromatic ring via hydrogen addition as well as catalytic cracking of the aliphatic chain occurs forming short-chain molecules. Further information regarding the changes is provided by FTIR, which shows that changes in the methylene-to-methyl ratio, a shift in the C–H asymmetric bending mode frequency, and a shift in aromatic ring vibration frequency indicates branching and substitution. The ^1^H and ^13^C NMR spectra confirmed the changes deduced from the IR spectra and in particular the reduction in aromatic character on hydrogenation. Detailed analysis produced by combining earlier assignments of NMR spectra of oils and approximate calculations of chemical shifts enabled a number of likely new structures to be proposed following hydrogenation. Comparison with cyclohexylnonadecane (CHND) shed light on the fact that along with saturation of the benzene ring, different conjugated substructures such as dienes and trienes are formed along with new branched molecules and their isomers. It is concluded that there is a need to use multiple chemical analysis techniques and tools to get a clearer picture of the changes that occur during hydrogenation. The changes that occur include changes in composition, i.e., reduction in aromatic content, and the formation of new molecules.

## Supporting Information Available

GC peak assignment based on NIST and Wiley library; detailed ^13^C NMR of PDD and its hydrogenated products; and CHND, 2D COSY of PDD, 8 h HC, and CHND (PDF)
