# Supplementary material for: Evaluation of Structural and Compositional Changes of a Model Monoaromatic Hydrocarbon in a Benchtop Hydrocracker Using GC, FTIR, and NMR Spectroscopy
Source: ACS Omega. 2023 Sep 18;8(39):35988–6000. doi: 10.1021/acsomega.3c03833 (PMC10552099; doi:10.1021/acsomega.3c03833)
Supplement: Supplementary file 1 — ao3c03833_si_001.pdf [file ao3c03833_si_001.pdf]

Evaluation of Structural and Compositional Changes of a Model Mono-aromatic Hydrocarbon in a Benchtop Hydrocracker Using GC, FTIR and NMR spectroscopy

Debashis Puhan*, Michael T.L. Casford, Paul B. Davies

Yusuf Hamied Department of Chemistry, University of Cambridge, Cambridge, United Kingdom
Lensfield Road, CB2 1EW
*dp617@cam.ac.uk

Table S1: GC Peak Assignment from Wiley and NIST Library

Retention time (minutes)	Library Match	Formula	Comments
2.476	Octylcyclohexane	C14H28	monocycloalkane
2.57	Heptacosane/Octacosane	C27H56/ C28H58	Straight chain paraffin
2.787	Cyclooctasiloxane		Stationary phase
2.896	1 Methylpropyl Cyclohexane/ 4 Methylpentyl Cyclohexane Dodecylcyclohexane Heptylcyclohexane 1,1-(1,3-Propanediyl)Bis-Cyclohexane/ 1,1-(1,2-Ethanediyl)Bis-Cyclohexane/ 1,1-(1,4-Butanediyl)Bis-Cyclohexane/ 1,1-(1,5-Pentanediyl)Bis-Cyclohexane/	C10H20	Mono/Bi cycloalkane with branching
3.411	1,1-(1,3-Propanediyl)Bis-Cyclohexane/ 1,1-(1,4-Butanediyl)Bis-Cyclohexane/		

	1,1-(1,5-Pentanediyl)Bis-<br>Cyclohexane/		
3.599	Octadecamethylcyclononasiloxane		Stationary phase
3.852	1 Methylbutyl Cyclohexane<br>2 Cyclohexyldodecane<br>2 Cyclohexyl Eicosane<br>1-Cyclohexylmethyl- 4ethyl<br>Cyclohexane<br>2 Cyclohexyl Undecane<br>2 Cyclohexyl Decane	C16H30<br>C15H28<br>C18H36<br>C11H22	Monocycloalkane with branching
4.023	Dodecylcohexane<br>Nonadecylcyclohexane<br>Octyl-Cyclohexane<br>4 Methylpentylcyclohexane<br>Nonylcyclohexane<br>Undecylcyclohexane<br>Decylcyclohexane<br>Pentadecylcyclohexane<br>Heptadecylcyclohexane<br>Heptylcyclohexane<br>Octylcyclohexane	C18H36	Monocycloalkane with branching
4.149	Dodecylbenzene<br>Undecylbenzene<br>Phenyldodecane<br>Tetradecylbenzene	C18H30	As supplied

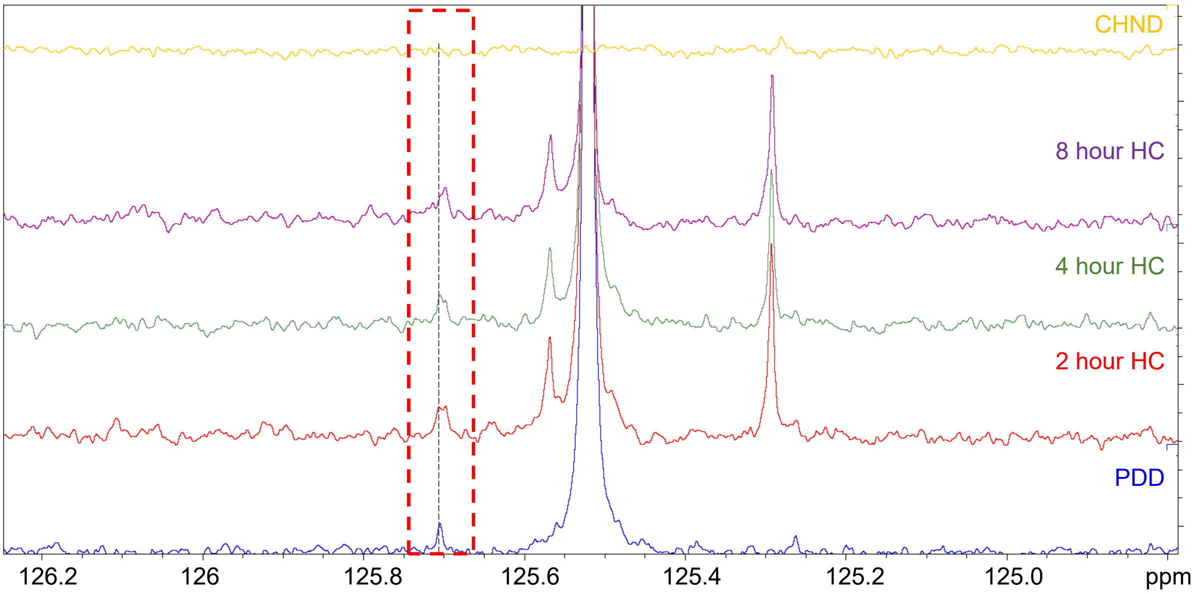

Figure S1: ^{13}C NMR of the samples in the aromatic region showing changes in the 125.7 ppm peak signal (dotted line) relative intensity.

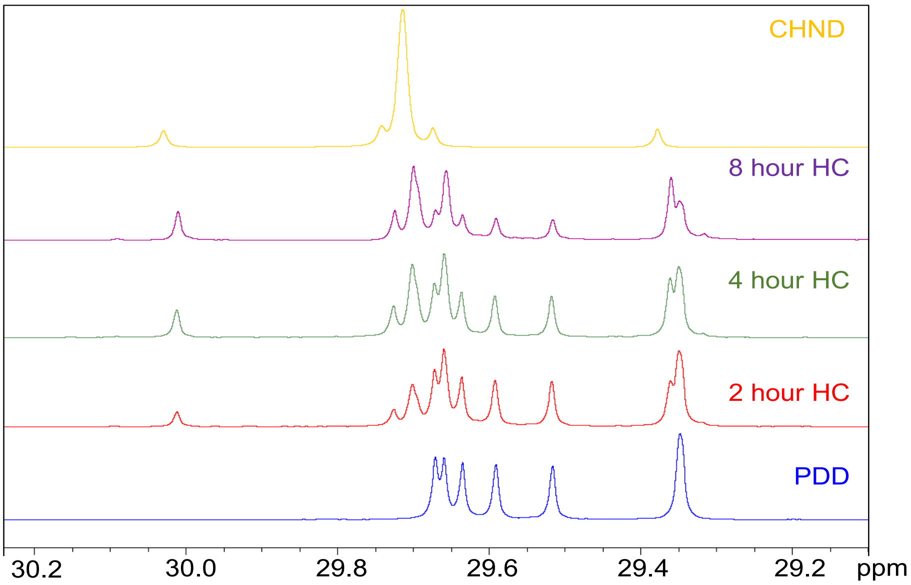

Figure S2: ^{13}C NMR of the samples in the aliphatic region showing detailed changes in peak signal relative intensity between 30.2 and 29.2 ppm.

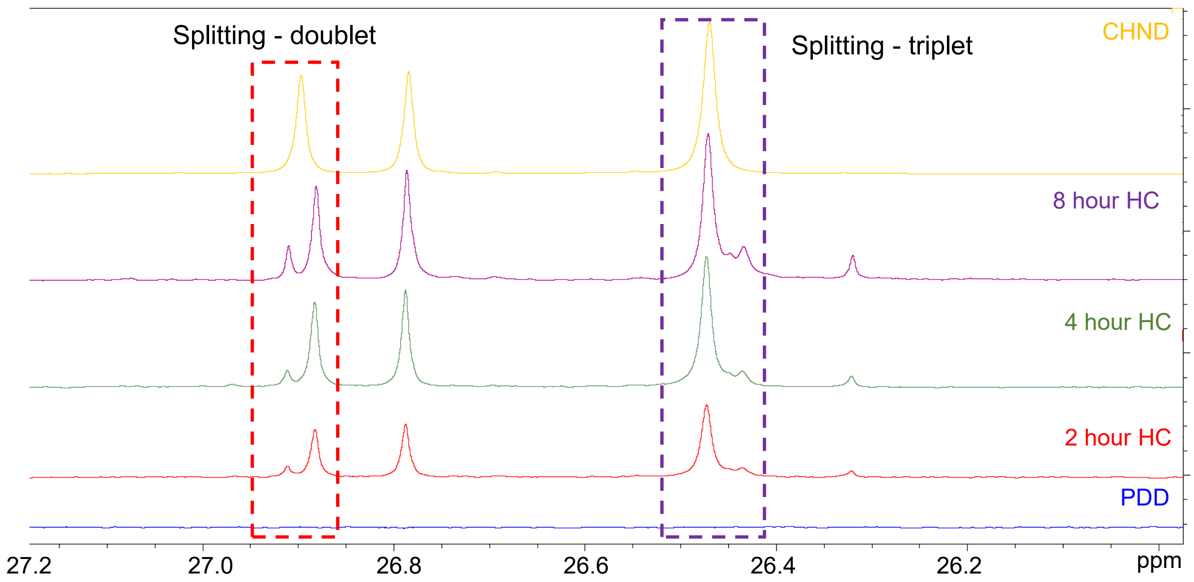

Figure S3: ^{13}C NMR of the samples between 27.2 and 26.2 ppm showing new peaks due to splitting.

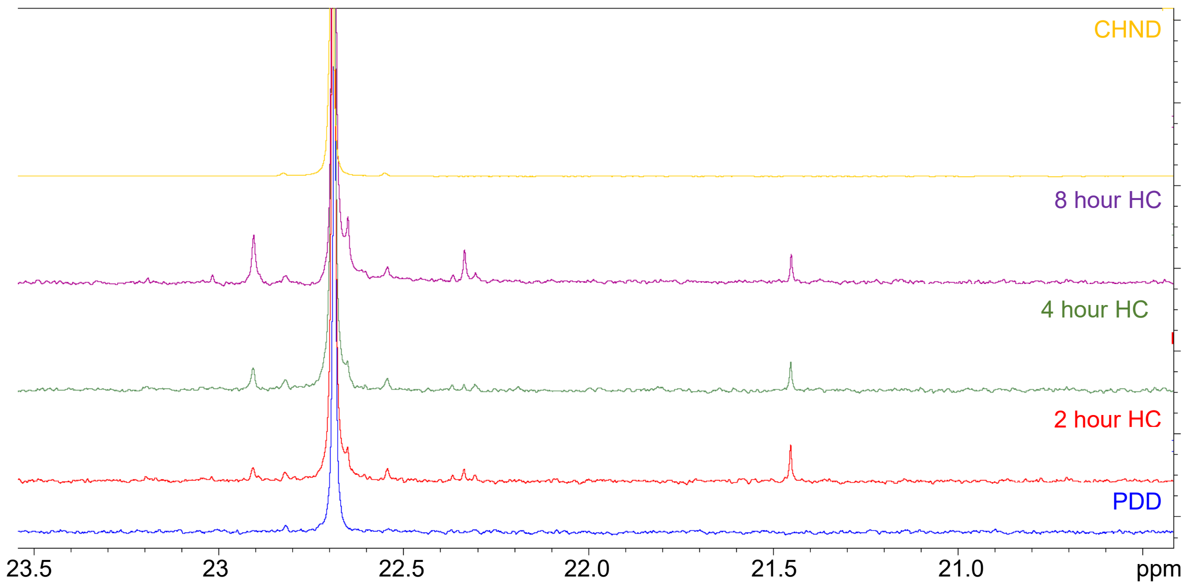

Figure S4: ^{13}C NMR of the samples in the aliphatic region showing new peaks between 23.5 and 21 ppm.

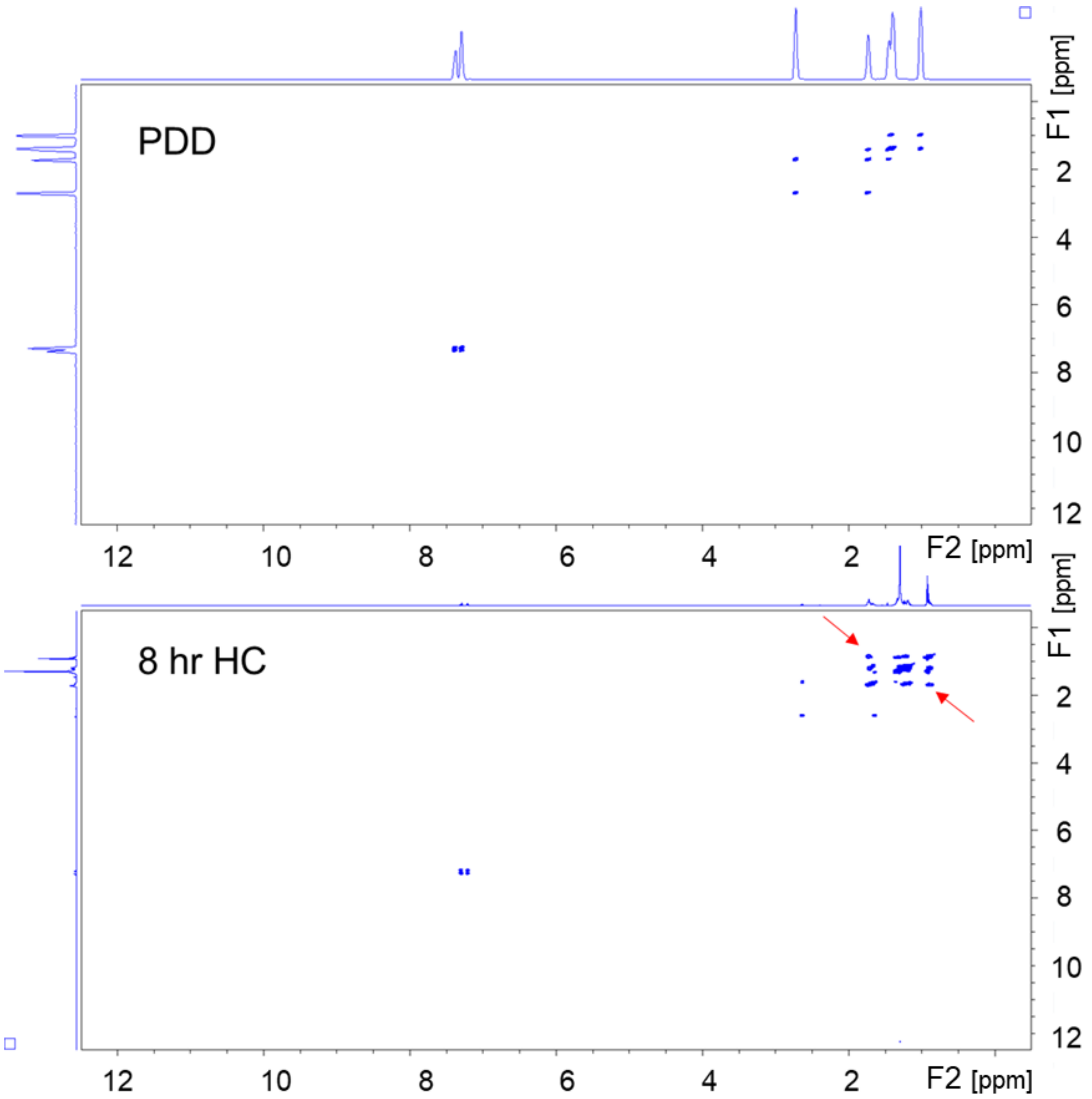

Figure S5: Comparison of Homonuclear Correlation Spectroscopy (COSY) showing spectral changes of 8-hour hydrogenated sample (8 hr HC) with respect to Phenyldodecane. Red arrows indicate the new hydrogen peaks that are coupled to each other.

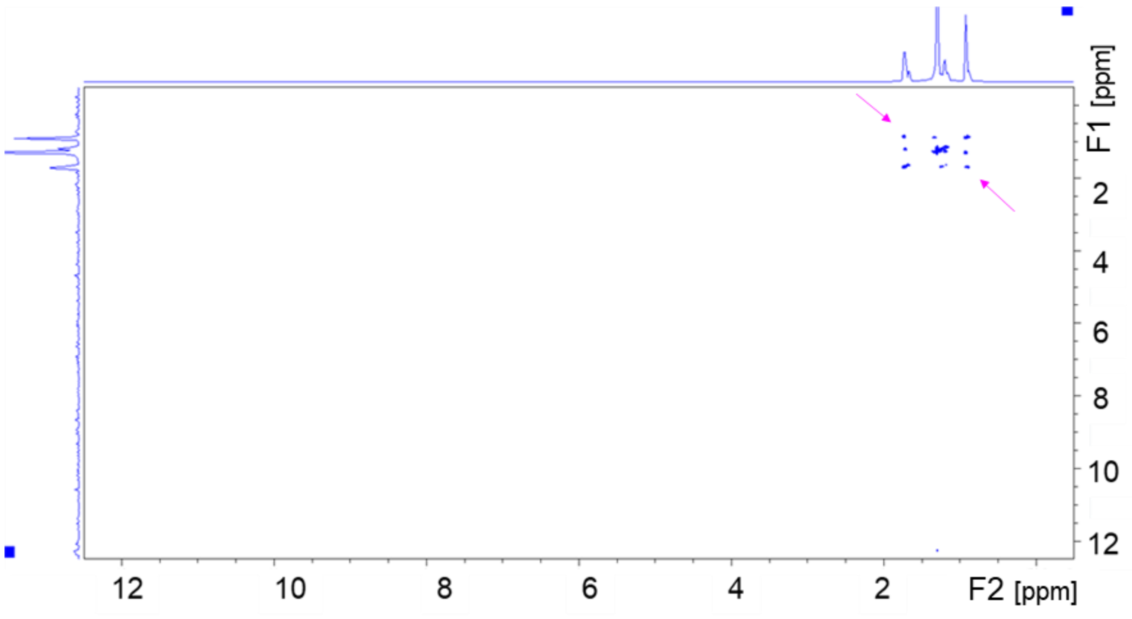

Figure S6: Homonuclear Correlation Spectroscopy (COSY) spectra of Cyclohexyl nonadecane (CHND).
